# Correlation of bioactive marker compounds of an orally applied *Morus alba* root bark extract with toxicity and efficacy in BALB/c mice

**DOI:** 10.3389/fphar.2023.1193118

**Published:** 2023-12-08

**Authors:** Julia Langeder, Mirijam Koch, Hannes Schmietendorf, Ammar Tahir, Ulrike Grienke, Judith M. Rollinger, Michaela Schmidtke

**Affiliations:** ^1^ Vienna Doctoral School of Pharmaceutical, Nutritional and Sport Sciences, University of Vienna, Vienna, Austria; ^2^ Division of Pharmacognosy, Department of Pharmaceutical Sciences, Faculty of Life Sciences, University of Vienna, Vienna, Austria; ^3^ Department of Medical Microbiology, Jena University Hospital, Jena, Germany; ^4^ Section of Experimental Virology, Department of Medical Microbiology, Jena University Hospital, Jena, Germany

**Keywords:** natural product, Mulberry Diels-Alder-type adducts, sanggenon, quantitation method, bioavailability, *in vivo*, lung, influenza

## Abstract

**Introduction:** In traditional Chinese medicine, the root bark of *Morus alba* L. is used to treat respiratory infections. Recently, anti-inflammatory and multiple anti-infective activities (against influenza viruses, corona virus 2, *S. aureus*, and *S. pneumoniae*) were shown *in vitro* for a standardized root bark extract from *M*. *alba* (MA60). Sanggenons C and D were identified as major active constituents of MA60. The aim of the present preclinical study was to evaluate, whether these findings are transferable to an *in vivo* setting.

**Methods:** MA60 was orally administered to female BALB/c mice to determine 1) the maximum tolerated dose (MTD) in an acute toxicity study and 2) its anti-influenza virus and anti-inflammatory effects in an efficacy study. A further aim was to evaluate whether there is a correlation between the obtained results and the amount of sanggenons C and D in serum and tissues. For the quantitation of the marker compounds sanggenons C and D in serum and tissue samples an UPLC-ESI-MS method was developed and validated.

**Results:** In our study setting, the MTD was reached at 100 mg/kg. In the efficacy study, the treatment effects were moderate. Dose-dependent quantities of sanggenon C in serum and sanggenon D in liver samples were detected. Only very low concentrations of sanggenons C and D were determined in lung samples and none of these compounds was found in spleen samples. There was no compound accumulation when MA60 was administered repeatedly.

**Discussion:** The herein determined low serum concentration after oral application once daily encourages the use of an alternative application route like intravenous, inhalation or intranasal administration and/or multiple dosing in further trials. The established method for the quantitation of the marker sanggenon compounds in tissue samples serves as a basis to determine pharmacokinetic parameters such as their bioavailability in future studies.

## 1 Introduction

Besides the coronavirus 2 which emerged in 2019 (SARS-CoV-2), influenza viruses and *Streptococcus pneumoniae* (pneumococci) belong to the most common pathogens causing pneumonia (Cilloniz et al., 2022; Dhoubhadel et al., 2022). Influenza is a respiratory illness with complications ranging from mild to severe symptoms or even death (Duwe et al., 2021). About three million severe cases of illness causing hospitalization occur annually worldwide (Paget et al., 2023) and the World Health Organization (WHO)^1^ estimates between 290,000 and 650,000 deaths resulting from seasonal influenza outbreaks. Factors that drastically increase mortality rates of influenza cases are bacterial co-infections with pathogens like *S. pneumoniae* (McCullers, 2014). The neuraminidases of influenza viruses and *S. pneumoniae* contribute to this lethal synergism (Walther et al., 2016). The dramatic economic and medical impact of this lethal interplay drives the intense search for new therapeutic interventions and curative drugs (Langeder et al., 2020).

*Morus alba* L., commonly called the white mulberry tree, belongs to the family of Moraceae. In traditional Chinese medicine and other Asian folk medicines, various parts of *M. alba* (leaves, fruits, twigs, and the root bark) are used for antiphlogistic, diuretic, expectorant, and antidiabetic properties (Batiha et al., 2023). Apart from these medicinal effects, white mulberry fruits are also used for their nutritive value since they are known to contain important proteins, carbohydrates, fiber, organic acids, vitamins, and minerals (Chen et al., 2021). Regarding respiratory infections, the root bark of *M. alba* (Sang Bai Pi in Chinese) is traditionally used for the treatment of cough, bronchitis, and other pulmonary diseases (Yadav et al., 2022).

**FIGURE 1:**
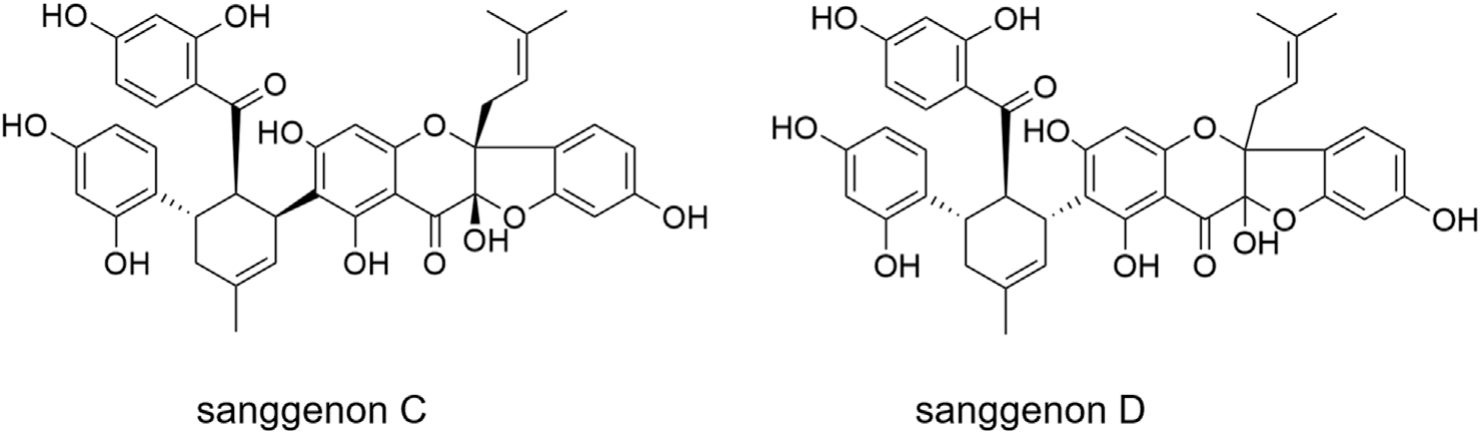
Major MDAAs contained in the *M. alba* root bark extract: sanggenon C and sanggenon D.

Recently, *M. alba* root bark has been shown to constitute promising dual inhibitors to combat viral and bacterial respiratory infections: it contains mulberry Diels-Alder-type adducts (MDAAs) which are prenylated flavonoids resulting from a [4 + 2]-cycloaddition of dehydroprenylphenols and chalcones (Grienke et al., 2016; Luo et al., 2021). In addition to the anti-infective properties, the anti-inflammatory potential of MDAAs has been identified *in vitro* (Rollinger et al., 2005; Chang et al., 2019). Recently, a specialized extract of the white mulberry root bark, labelled as MA60, enriched in 29% MDAAs (6.9% of sanggenon C and 10.7% of sanggenon D) was shown to exhibit promising anti-infective properties against influenza viruses, SARS-CoV-2, *Staphylococcus aureus*, and *S. pneumoniae* (Langeder et al., 2023; Wasilewicz et al., 2023). The high MDAA content clearly correlated with a dual inhibitory activity against influenza viruses and *S. pneumoniae* (Langeder et al., 2023). The two major MDAAs, sanggenons C and D (Figure 1), contained in the root bark, show remarkable anti-influenza virus, anti-*S. pneumonia* and anti-*S. aureus* activities *in vitro* (Grienke et al., 2016). These results indicate sanggenons C and D as valuable bioactive marker compounds for preclinical studies.

*In vitro* and *ex vivo* models are approaches to identify bioactive lead compounds. However, the translatability to an *in vivo* setting or even clinical studies is challenging^2^. Aspects like toxicity or complex pathogen-host interactions like the cellular and humoral immune defense of mammals are completely neglected in an *in vitro* setting (Roosenhoff et al., 2018). Thus, preclinical studies are essential (Butterweck and Nahrstedt, 2012). Mouse models of influenza A virus infection are valuable to study therapeutic effects (Barnard, 2009).

To translate the promising *in vitro* data of MA60 to an *in vivo* setting, the present study aimed to determine the maximum tolerated dose (MTD) of MA60 in female BALB/c mice (acute toxicity study) to be then used in the subsequent influenza virus infection model (efficacy study). These preclinical studies aim i) to evaluate the potential toxicity and to identify the most suitable oral dosage for the antiviral *in vivo* study, ii) to study whether an oral application ensures anti-influenza and/or anti-inflammatory effects, and iii) to determine the concentration of two selected marker constituents (sanggenons C and D) in serum as well as tissue samples after oral application of MA60.

## 2 Material and methods

### 2.1 Plant material, standards and MA60 extract preparation

The plant material from *M. alba* (root bark, batch no. 460797) was purchased from Plantasia in 2018. A voucher specimen (JR-20190928-A1) is deposited at the Department of Pharmaceutical Sciences, Division of Pharmacognosy, University of Vienna, Austria.

Double distilled water, HPLC-grade acetonitrile (VWR) and formic acid (VWR) were used for the chromatographic separation. For extract preparations, petroleum ether and n-hexane were distilled according to the Austrian Pharmacopoeia. Isopropanol (VWR) was purchased in analytical grade. Prior to LC-MS analyses, samples were dissolved in HPLC-grade methanol (VWR).

The MA60 extract was prepared as previously reported (Langeder et al., 2023). Briefly, a two-step extraction method was performed on a Dionex ASE 350 accelerated solvent extraction (ASE) instrument from Thermo Fisher Scientific, United States. Approximately 15 g of ground *M. alba* root bark were filled in a 34 mL extraction cell. In a first defatting step, the root bark was extracted with n-hexane in the flow mode (120°C) to remove highly lipophilic compounds. The obtained n-hexane extract was discarded. The second and essential extraction step was carried out with a mixture of isopropanol and petroleum ether in the ratio 2:1 in the flow mode (80°C). This procedure was repeated multiple times to obtain 2.8 g extract from 300 g plant material. MA60 consists of 10.7% sanggenon C and of 6.9% sanggenon D. Further, it contains other sanggenons, i.e., sanggenon B (1.2%), sanggenon G (1.2%), sanggenon O (0.8%) and sanggenon E (1.0%).

Sanggenons C and D were initially isolated from a methanolic *M. alba* root bark (2 kg) extract. Eleven fractions were obtained via flash chromatography (Interchim puriFlash^®^ 4250) separated on a Puriflash 25 Silica HC 200G 25 µm column. Fractions 5 and 6 were further purified on a Gemini-Nx C_18_ phemomenex^®^ column yielding 515 mg of sanggenon C (purity: 99%) and 730 mg of sanggenon D (purity: 99%), as previously described in detail (Langeder et al., 2023).

### 2.2 Animals, cells, and virus

Female mice are known to be more susceptible to influenza virus infection than male mice (Robinson et al., 2011). Therefore, female BALB/c mice (eight-weeks-old; 16–18 g; n = 66) were used in the present *in vivo* studies. Mice were purchased from Charles River (Bad Sulzfeld, Germany). They were housed in individually ventilated cages, at 22°C ± 2°C with a relative humidity of 55% ± 10% and a 14/10 h light/dark cycle. Mice were given food and water *ad libitum*.

Madin Darby canine kidney (MDCK) cells (Friedrich Loeffler Institute, Riems, Germany) were applied in Eagle’s minimal essential medium supplemented with 2 μg/mL trypsin, 2 mM L-glutamine, and 1% nonessential amino acids.

Isolation and propagation of the working passage of influenza virus A(H1N1)pdm09 HA-G222-mpJena/5258 were published (Seidel et al., 2014).

### 2.3 Ethics statement

All trial procedures and animal care activities were conducted following the German Animal Protection Law. Experiments were approved by the Thüringer Landesamt für Verbraucherschutz (Reg.-Nr.: UKJ-18-015).

### 2.4 Dose finding study and acute toxicity of MA60 *in vivo* (experiment I)

For the development of the standard operating procedure (SOP) for the preparation of MA60 suspensions, the solubility of MA60 was evaluated. Based on previous studies from literature, 0.3% carboxymethyl cellulose (CMC) was used as a vehicle for preparing an MA60 extract suspension for oral application (Lim et al., 2013; Yimam et al., 2017). The 300 mg/kg dose was selected as maximum dose for the acute toxicity study. This was the maximum dose still resulting in a nearly homogenous solution (Supplementary Figure S1A) which is the prerequisite for oral gavage.

Aiming to define the dose and to exclude/reduce the risk of unacceptable side effects for experiment II (efficacy study), an acute toxicity study was performed. Twenty mice were randomly divided into four groups of five mice. One group was treated with the solvent (placebo: 0.3% CMC) and the other groups with 30, 100, and 300 mg/kg of solved MA60 (Supplementary Figure S1B). According to our previous results, influenza virus replication decreases until day 7 after infection (Seidel et al., 2014). The time schedule for MA60 administration was selected based on these observations. For the administration of MA60, oral gavage once daily was applied based on promising literature data reported for an ethanol extract from *M*. *alba* rootbark in 0.3% CMC applying 200 or 400 mg/bw/daily in a mouse model of LPS-induced airway inflammation (Lim et al., 2013).

Body weight and clinical score (weight change, physiognomy, and behavior) of mice were determined daily and considered for the determination of the MTD. Mice were sacrificed and organ tissue samples (lung, liver, heart, spleen) were aseptically removed and weighed 3 hours after the last treatment. In addition, serum was obtained from collected blood samples by centrifugation at 855 *g* for 15 min. The lung, liver, spleen, and serum samples of mock-infected mice were also used to establish and validate a UPLC-MS method for the quantitation of the marker compounds sanggenon C and D.

### 2.5 Efficacy study regarding anti-influenza and anti-inflammatory effects of MA60 *in vivo* (experiment II)

To determine the antiviral effect of MA60 *in vivo*, 46 mice were randomly divided into experimental groups as summarized in Supplementary Table S1. Mice were treated orally (per oral gavage) once daily for a maximum of 7 days with either the solvent (placebo: 0.3% CMC; n = 23) or 100 mg/kg of solved MA60 (n = 23). One hour after the first application of MA60, groups of mice were mock-infected (NaCl) or infected with the influenza virus in NaCl. For this procedure, mice under isoflurane anesthesia were inoculated intranasally with 20 µL NaCl or 10^5^ TCID_50_/20 µL of HA-G222-mpJena/5258 diluted in NaCl. The selected number of mice as well as the statistical evaluation are based on literature data (Richardson and Overbaugh, 2005).

Body weight and clinical score (weight change, physiognomy, and behavior) of mice were determined at least once daily. Mice that lost more than 20% of their initial body weight over 48 h or 25% of their initial body weight and/or became severely ill were sacrificed for ethical reasons (n = 3; two placebo-treated, influenza virus-infected mice and one MA60-treated, influenza virus-infected mouse).

One hour after the second treatment (day 1 p.i.), as well as 24 h after the last treatment (day 7 p.i.), groups of mice were sacrificed and organ tissue samples (lung, liver, spleen) were aseptically removed. Serum was obtained from collected blood samples by centrifugation at 855 *g* for 15 min. The superior lobe of influenza virus-infected right lung was homogenized in test medium and used for virus titer determination (Reed and Muench, 1938). Another right lung lobe was frozen for analysis of cytokine mRNA levels as described in 2.6. The left lobe was fixed in 10% formalin and embedded in paraffin for histopathological evaluation. For that, sections of 4 µm were prepared and stained with hematoxylin and eosin. Further two right lung lobes, liver, spleen, and serum of mock-infected mice were used for the determination of the content of sanggenons C and D.

### 2.6 RNA isolation, reverse transcription and quantitative PCR

Lung samples were homogenized in lysis buffer and TissueLyzer beads (Qiagen, Hilden) in the TissueLyzer II (Qiagen, Hilden). Then RNA was isolated with the RNeasy^®^ Mini Kit (Qiagen, Hilden) according to the producer’s manual. NP-vRNA copy number was determined after reverse transcription of 1,000 ng RNA with uni12 primer (Hoffmann et al., 2001) by a quantitative PCR with two NP-gene targeting primers and a NP-plasmid standard. The expression profile of cytokines (IP10, IL-6, IFN-ß, CXCL9 and Eif2ak2) was determined after reverse transcription of 1,000 ng RNA with oligo dT primer by a semi quantitative PCR with cytokine mRNA-targeting primers. The expression of GAPDH mRNA was used as a baseline control. Results are calculated and expressed as Log2(2^-ΔΔCt)-fold change of mRNA expression related to control (Pfaffl et al., 2002). Primer sequences and PCR temperature profiles are summarized in Tables (Supplementary Tables S2–S4).

### 2.7 Statistical analysis of *in vivo* data

Semi-quantitative PCR results were analyzed using EXCEL 2016 software according to the method described by Pfaffl (Pfaffl, 2004). Data are presented as mean with standard deviation (SD) calculated with EXCEL 2016.

Statistical analysis of data is based on “basic statistical considerations in virological experiments” published by (Richardson and Overbaugh, 2005). The number of animals (n) per experimental group is given in Supplementary Figure S1 (acute toxicity study) and Supplementary Table S1 (efficacy study). Mice succumbing to infection before day 7 are mentioned in paragraph 2.5 and 3.2. After performing normality test (Shapiro-Wilk) and equal variance test (Brown-Forsythe), the significance of differences within multiple cardinal data sets (body weight, cytokine mRNAs) were analyzed with One-way ANOVA (normal distribution) or Kruskal–Wallis One Way Analysis of Variance on Ranks (non-normal distribution) with Sigmaplot 14.0. Virus titers and nucleoprotein gene copies of the mock-treated, influenza virus-infected and the MA60-treated influenza virus-infected group of mice were analyzed with the unpaired, two-sided Student’s t-test by using EXCEL 2016. *p* ≤ 0.05 was set as cut off for statistical significance.

### 2.8 Serum and tissue extraction protocol

The bio sample extraction was adapted according to the published workflow from Yuan and co-workers (Yuan et al., 2012). 50 μL of thawed serum sample was transferred to a 1.5 mL Eppendorf tube. 1,000 μL of MTBE was added and shaken for 1 h. After a centrifugation step at 13,400 rpm for 15 min, 900 µL of supernatant was removed and the solvent was evaporated until complete dryness.

To analyze tissue samples, an aliquot of approximately 200 mg of each mouse liver, the whole spleen and lung were homogenized in a Zentrimix 380R (Hettich) at 4°C for 15 min at 1,500 rpm in a separate tube by three zirconium oxide beads (Ø 3 mm) in 500 µL water. 900 μL cooled methanol (−70°C) was added and ultrasonicated for 5 min in an ice bath. After vortexing, the samples were incubated for 1.5 h at −70°C. Then, tissue homogenates were centrifuged at 15,000 rpm in a refrigerated centrifuge (4°C) for 10 min. Supernatants were collected and the incubation cycle was repeated for exhaustive extraction with 400 µL fresh methanol (−70°C) followed by ultrasonication (10 min on ice) and vortexing under cooled conditions (4°C). Samples were transferred to a refrigerator for a 30 min incubation at −70°C. After centrifugation, supernatants were combined and dried in a GeneVac EZ-2 plus using the program “Aqueous” set to 30°C. For analysis, serum, as well as tissue samples, were dissolved in 50 µL MeOH, centrifuged and 5 µL were injected in duplicates for analysis.

### 2.9 UHPLC-ESI-MS analysis and method validation for the quantitation of marker compounds sanggenon C and sanggenon D in serum and tissue samples

UHPLC-ESI-MS (Thermo Fisher Scientific, CA) analysis was performed on a Dionex UltiMate 3,000 system coupled to an LTQ XL ion trap mass spectrometer. MS detection was carried out using HESI source (300°C heater temperature, 40/10/1 arb. units for the sheath, aux and sweep gases, respectively and 3.5 kV spray voltage at 275°C capillary temperature) to achieve negative ion mode ionization. For quantitation, SIM (single ion monitoring) was applied in the negative mode. The [M-H]^-^ adduct 707.21 Da was used as a center mass with a 1.00 Da window. For confirmation and method validity, selective MS/MS scans of the 3 most abundant ions were achieved through collisional induced dissociation (CID) fragmentation at 30% normalized collision energy.

Separation was carried out on a Waters ACQUITY BEH phenyl column (2.1 × 100 mm, 1.7 µm) within 13 min. Solvent A: Water/formic acid (99.9:0.1), Solvent B: Acetonitril/formic acid (99.9:0.1). The following gradient was applied: 0–2 min: 30% B, 2–13 min: 30%–98% B, followed by a washing step: 13–17 min 98% B and an equilibration step for 3 min 5 μL of dissolved sample in methanol were injected. Data acquisition and evaluation was carried out using Xcalibur (version 4.27.0.19). The UHPLC-ESI-MS method was validated for specificity, linearity, precision, and extraction recovery according to ICH guidelines^3^. In terms of specificity, chromatograms of the spiked samples were compared to respective blank serum or liver samples. Calibration curves were established by serial dilution of standards of sanggenons C and D, respectively in a ratio of 1:3 in methanol (level 0: 1 mg/mL) followed by injection of each level in triplicate. The peaks were integrated using Thermo Xcalibur software using the integration algorithm ICIS. Limit of detection (LOD) and limit of quantitation (LOQ) were evaluated visually as a signal-to-noise ratio of more than 3 times (LOD) or 10 times (LOQ). Linearity was determined as the range of all included calibration levels in the regression equation. For precision, intraday (within 1 day, sample #64) and interday (over 3 days, sample #65) measurements were evaluated accordingly. To assess extraction recovery, spiking experiments were conducted as follows: Stock solutions of the extract MA60 were prepared at a concentration of 1 mg/mL. 10 μL of the stock solution was added to 50 µL of blank serum or blank liver homogenate and treated as described above. By integration of the AUC of the peak of sanggenon C (serum samples) and sanggenon D (tissue samples) and comparing it to the peak area of sanggenons C and D, respectively in a 1:5 dilution of the stock solution, the recovery rate was calculated accordingly.

**FIGURE 2:**
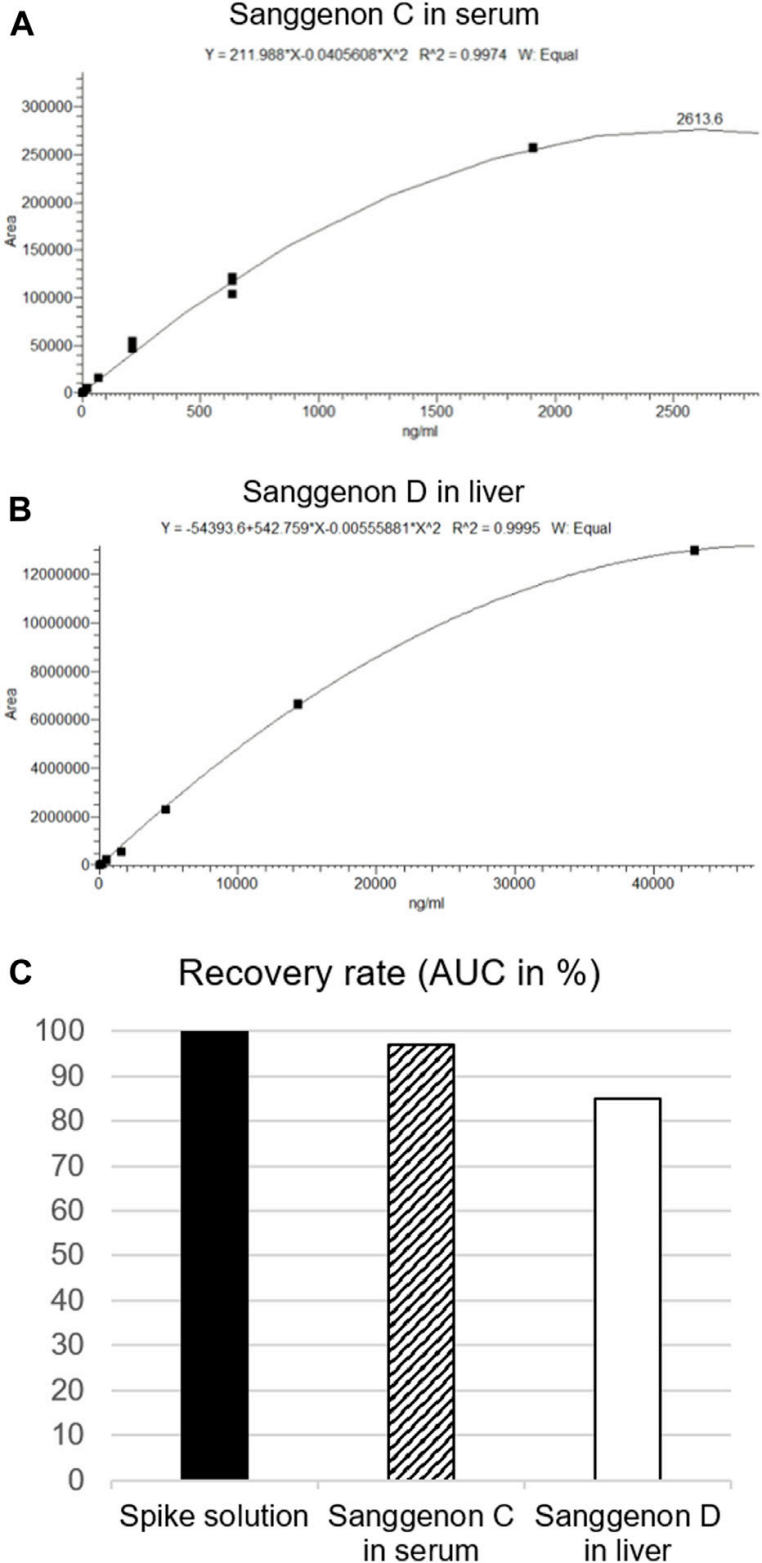
Calibration curves, fitting and dilutions of **(A)** sanggenon C for serum samples and **(B)** sanggenon D for liver samples. **(C)** Extraction recovery rates given as % of AUC of sanggenon C and D from the respective biomatrix in comparison to the AUC from the spike solution (set to 100%).

MA60 contains a well-characterized amount of bioactive MDAAs of 29% (Langeder et al., 2023). Sanggenons C and D calibration curves were obtained by serial dilution down to 1.88 ng/mL (Figure 2). A quadratic fit of the calibration curves revealed correlation coefficients of *R* ^2^ ≥ 0.9974 (Table 1). Validation parameters (specificity, linearity, precision, and extraction recovery) were evaluated in accordance with ICH guidelines.^4^ The specificity was assessed by comparing the selected MS/MS spectra of serum and tissue samples to the fragment spectra of sanggenons C and D. As there was a precise matching of the standard fragment ions to the fragment ions found in serum and tissue samples, it can be concluded that the established method is specific for the marker compounds (Supplementary Figure S2).

**TABLE 1:** Validation parameters for the quantitation of sanggenon C in serum samples and sanggenon D in liver samples.

Compounds	Sanggenon C	Sanggenon D
Regression equation (*y =*)	211.988x-0.0405608x^2^	−54393.6 + 542.759x-0.00555881x^2^
Correlation coefficient (*R* ^*2*^)	0.9974	0.9995
Linearity range (ng/mL)	2.62–1,906.72	2.1–42,962
LOD (ng/mL)	0.87	3.92
LOQ (ng/mL)	2.91	13.05
Intraday precision (%)	n.d.	6.20
Interday precision (%)	n.d.	7.71
Extraction recovery (%)	95	85

n.d., not determined.

The extraction efficiency for serum and tissue samples was probed by performing spiking experiments. 10 μL of a stock solution of MA60 (1 mg/mL) was spiked to samples of blank serum and liver. Sanggenons C and D were extracted according to the given protocol. Their peak areas were compared to the extract solution (diluted 1:5), respectively. From the spiked samples, 97% of sanggenon C in serum samples and 85% of sanggenon D in liver samples were retrieved. These results confirmed the suitability of the extraction protocol for sanggenon C in serum samples and sanggenon D in liver samples (Supplementary Figure S2). Interestingly, the extraction recoveries for sanggenon D in serum samples (84%) and sanggenon C in liver samples were significantly lower (26%), and therefore not applied as marker compounds in the respective matrices.

## 3 Results

### 3.1 Dose finding study and acute toxicity of MA60 *in vivo* (experiment I)

**FIGURE 3:**
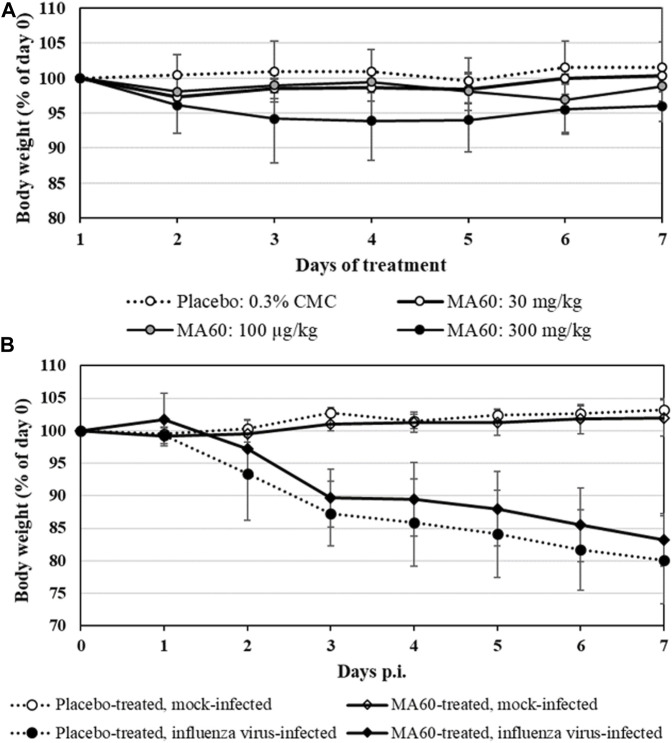
Mean body weight changes of 8-week-old female BALB/c mice which were treated **(A)** once daily for 7 days with 30, 100 or 300 mg/kg MA60 or placebo (solvent: 0.03% CMC) orally in the toxicity study or **(B)** once daily for 7 days with 100 mg/kg MA60 or placebo (solvent: 0.03% CMC) orally in the efficacy study. Treatment started 1 h before intranasal mock-infection (NaCl) or infection with influenza virus A(H1N1)pdm09 (10^5^ TCID_50_/mouse in 20 µL NaCl).

BALB/c mice were treated per gavage feeding with 30, 100, and 300 mg/kg of MA60 in 0.3% CMC or 0.3% CMC (placebo) once daily for 7 days to determine the MTD for the subsequent *in vivo* efficacy study. Daily monitoring of body weight revealed that neither treatment with 30 nor with 100 mg/kg of MA60 caused a significant body weight change compared to placebo (Figure 3A; Supplementary Table S5). In contrast, mice treated with 300 mg/kg of MA60 continuously lost body weight until day 4 of treatment (*p* = 0.03 at day 4 of treatment compared to the placebo-treated group). The 300 mg/kg dose of MA60 did not affect the weight of liver, lung, heart, and spleen (Supplementary Table S6). The applied tests and the results of statistical analysis of the percentage of daily body weight (% of day 0) are summarized in Supplementary Table S5. After day 4, a slight amelioration was observed.

In summary, body weight loss was the only adverse effect observed after treatment (clinical score not shown). The 300 mg/kg dose of MA60 causes more than a 10% depression in body weight gain. Thus, the 100 mg/kg dose of MA60 was identified as MTD in the acute toxicity study.

### 3.2 Efficacy study regarding anti-influenza and anti-inflammatory effects of MA60 *in vivo* (experiment II)

**FIGURE 4:**
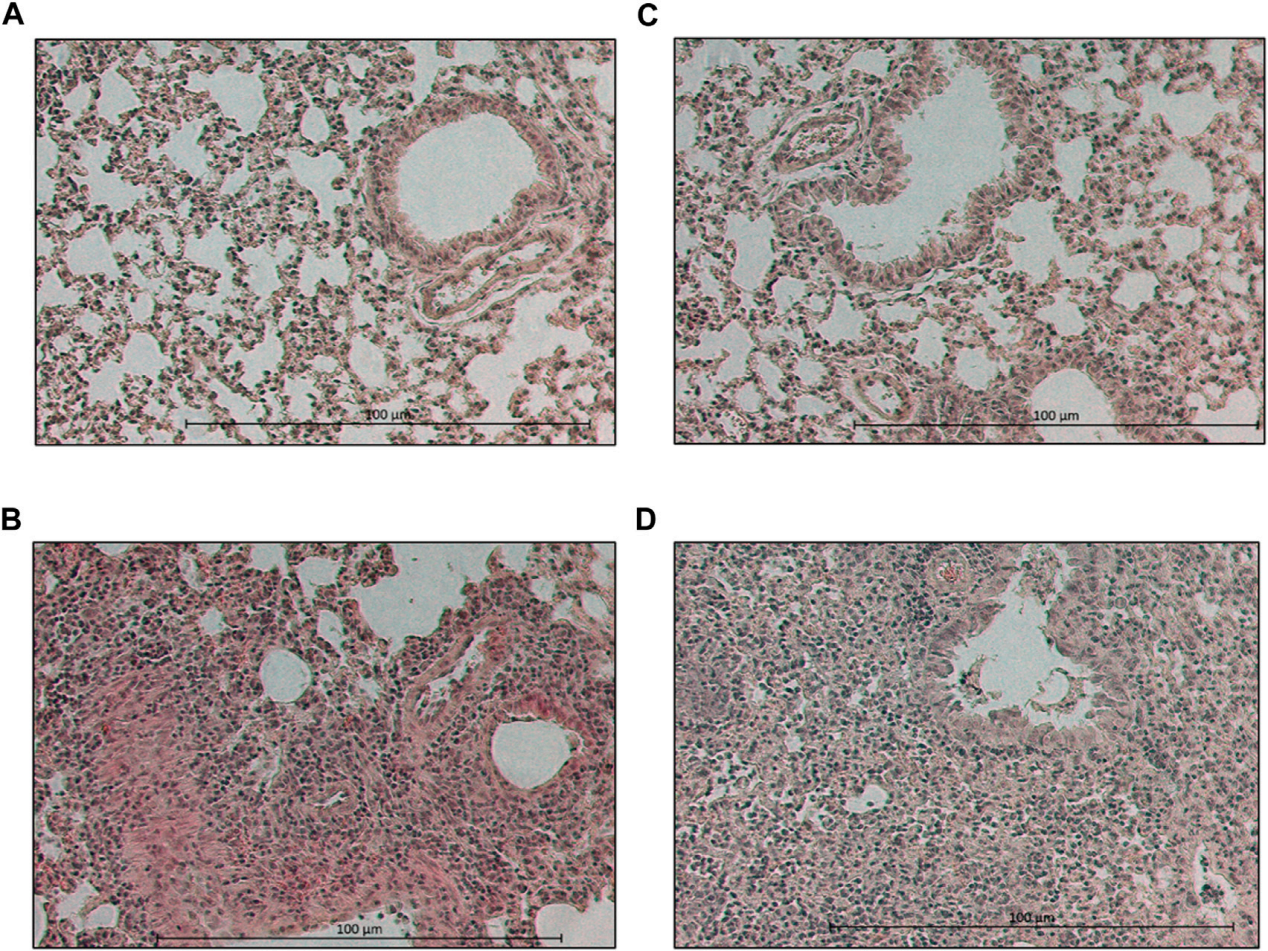
Photographs of hematoxylin-eosin-stained lung sections of day 7 p.i. Lung section of a **(A)** placebo-treated, mock-infected, **(B)** placebo-treated, virus-infected, **(C)** MA60-treated, mock-infected, and **(D)** MA60-treated, influenza virus-infected BALB/c mouse is shown, for example,.

Based on the dose-finding experiment, the 100 mg/kg dose of MA60 was well tolerated when administered orally once a day for 7 days to 8-weeks-old female BALB/c mice (MA60-treated, mock-infected). Neither body weight (Figure 3B) nor lung histology (Figures 4A,C and Supplementary Figure S3A, S3C) and cytokine mRNA expression (Figure 6) were significantly affected when applying this MA60 dose (Supplementary Tables S7, S8).

**FIGURE 5:**
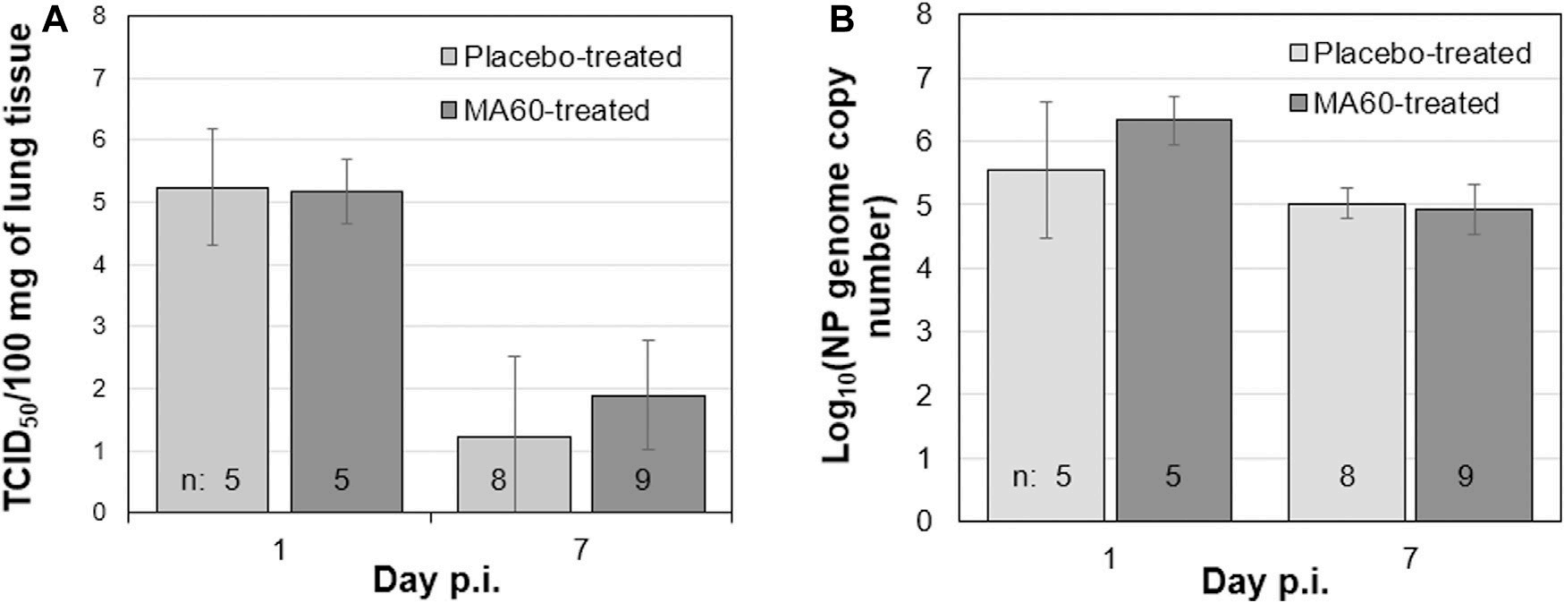
Oral treatment with 100 mg/kg MA60 (7 days, once per day) did not inhibit influenza virus replication in the lung. To study the effect of MA60 on viral replication, **(A)** virus titers and **(B)** nucleoprotein genome copies were determined in lung tissue samples.

**FIGURE 6:**
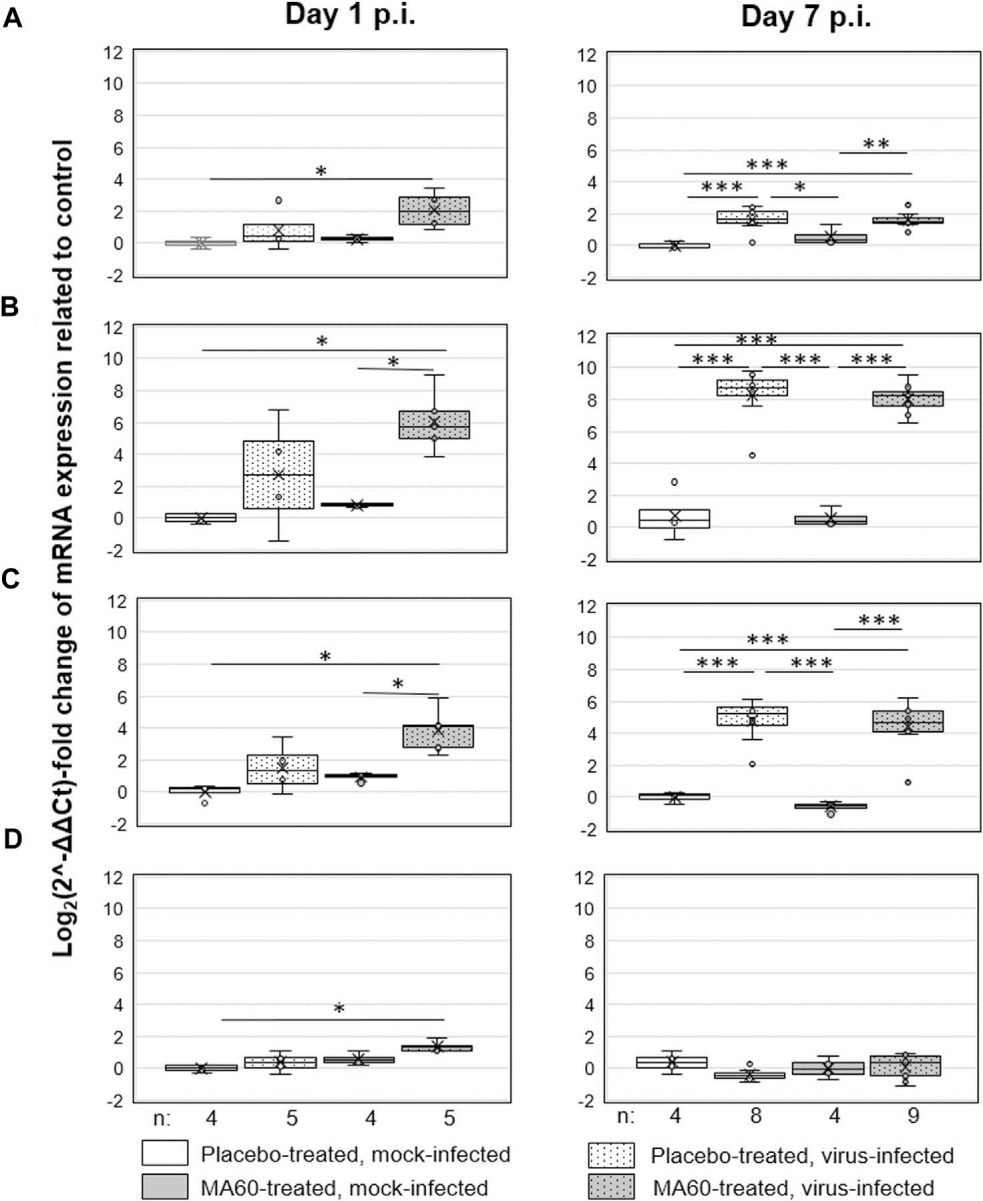
Oral treatment with 100 mg/kg MA60 (7 days, once per day) had no significant effect on cytokine expression in the lung of influenza virus-infected BALB/c mice. The mRNA copies of **(A)** IL-6, **(B)** IP10, **(C)** CXCL9, and **(D)** IFN-ß were determined by qPCR.

In contrast, placebo-treated, influenza virus-infected mice lost body weight starting on day 2 after infection (Figure 3B). Due to critical body weight loss, one mouse of this experimental group had to be sacrificed for ethical reasons on day 5 p.i. and another one on day 6 p.i. As of day 2 p.i. or day 3 p.i., the difference in body weight was statistically significant compared to placebo-treated, mock-infected mice (*p* values between <0.001 and 0.020) or MA60-treated, mock-infected mice (*p* values between <0.001 and 0.027), respectively. The applied statistical tests and the calculated *p* values are summarized in Supplementary Table S7. High virus titers and nucleoprotein gene copy numbers were detected on day 1 p.i. (Figures 5A, B). Infectious virus was nearly eliminated on day 7 after virus infection (Figure 5A) whereas nucleoprotein gene copies declined more slowly (Figure 5B). The expression of cytokines was enhanced, however not significantly on the day after influenza virus infection when comparing placebo-treated, mock-infected mice with placebo-treated, influenza virus-infected mice (Figure 6; Supplementary Table S8). In contrast, a significant difference was found between both experimental groups for the expression of IL-6, IP10, and CXCL9 7 days after virus infection (Figure 6; Supplementary Table S8).

The loss of body weight occurred more slowly and was less pronounced in MA60-treated, influenza virus-infected mice compared to placebo-treated, influenza virus-infected mice (Figure 3B). The body weight differences were statistically different between day 3 and 5 p.i. (*p* values between 0.022 and 0.004) or day 4 p.i. (0.004) when comparing MA60-treated, influenza virus-infected mice with placebo- or MA60-treated, mock-infected mice, respectively. However, the observed difference between MA60-treated, influenza virus-infected mice and placebo-treated, influenza virus-infected mice was not significant (Supplementary Table S7). Only one mouse of this experimental group had to be sacrificed for ethical reasons on day 6 p.i. The treatment effect of 100 mg/kg MA60 administered orally was not strong enough to significantly reduce virus titers (Figure 5A) and the number of viral nucleoprotein genome copies (Figure 5B). At day 1 after infection, the cytokine expression (IL-6, CXCL-9, IP10, and IFN-ß) in lung samples of MA60-treated, influenza virus-infected mice was stronger than in all other experimental groups. The difference was not significant in comparison to the placebo-treated, influenza virus-infected group but it was significant in comparison to the placebo-treated, mock-infected and/or MA60-treated, mock-infected groups (Figure 6; Supplementary Table S8). The comparison of lung histopathology did not reveal differences between placebo-treated and MA60-treated influenza virus-infected mice (Figures 4B, D; Supplementary Figures S3B, S3D).

### 3.3 Quantitation of sanggenons C and D in serum and tissue samples using UPLC-ESI-MS

**FIGURE 7:**
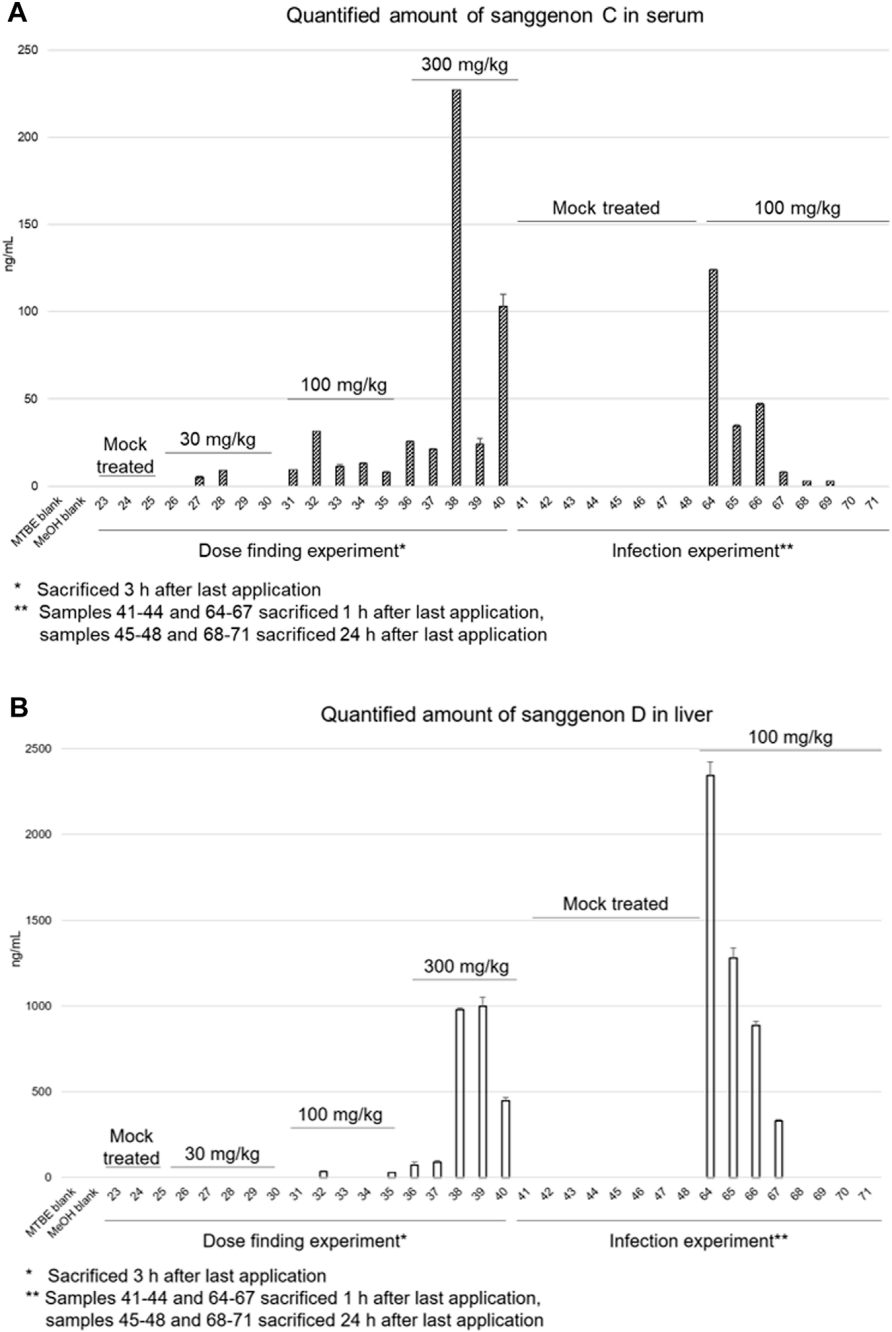
Quantitative results of sanggenon C in serum samples **(A)** and sanggenon D in liver samples **(B)** for the dose finding trial where 30 mg/kg, 100 mg/kg and 300 mg/kg extract (MA60) were applied orally and the infection trial with 100 mg/kg body weight, respectively.

In the samples from the acute toxicity trial, a dose-dependency could be observed for the content of sanggenon C in serum samples (Figure 7A). In the lowest dose group (30 mg/kg) an amount of sanggenon C below 9.10 ng/mL was found; in the 100 mg/kg dose group between 7.94 ng/mL and 31.53 ng/mL, and in the group of mice treated with 300 mg/kg of MA60 between 21.21 ng/mL and 227.26 ng/mL of sanggenon C were determined (Supplementary Table S9).

In the efficacy trial (100 mg/kg dose), serum concentrations of sanggenon C were found to be between 8.00 ng/mL and 124.22 ng/mL (#64-#67) after 1 h. After 24 h, the concentrations were below 2.77 ng/mL or not detectable (#68-#71).

The amount of sanggenon D was determined in both the acute toxicity as well as the efficacy experiment in lung, liver, and spleen samples (see Figure 7B; Supplementary Table S9). In lung samples, sanggenon D was detected in relatively high amounts between 343 and 10,306 ng/mL in four samples (#29, #32, #33 and #35). In liver samples of the low dose group sanggenon D was not detected. Only in two (#32 and #35) out of five samples, a sanggenon D concentration of 34.18 ng/mL and 26.97 ng/mL was detected, respectively. However, in the high dose group (300 mg/kg) especially in liver tissues derived from #38 and #39, a concentration of almost 1 µg/mL was determined. In general, samples from mice #38-#40 resulted in high concentrations of sanggenon C (serum) and sanggenon D (liver). Comparable to the serum concentrations of sanggenon C in the infection experiment, a high amount of sanggenon D was detected when mice were sacrificed after 1 h. In contrast, sanggenon D could not be determined after 24 h in the liver. None of the marker compounds was found in spleen samples (data not shown).

## 4 Discussion

The presented *in vivo* studies i) enabled the determination of the MTD of MA60, ii) gave first insights into the potential efficacy of MA60 against influenza virus infection *in vivo*, and iii) allowed for the correlation of marker compound concentrations in serum and tissue samples with the doses of MA60 applied orally.

The MTD of MA60 was determined in female BALB/c mice by oral application of 30, 100, and 300 mg/kg MA60 in the acute toxicity study using the following parameters: body weight, clinical score, and organ weight (lung, liver, heart, and spleen). Higher doses, published in two mice studies with ethanolic root bark extracts of *M. alba* (Lim et al., 2013; Yimam et al., 2019), did not reveal homogeneous solutions of MA60 in 0.3% CMC. Thus, the dose limit for acute toxicity studies of 1,000 mg/kg/day for rodents as recommended in the ICH guideline M3(R2)^5^ could not be applied for practical reasons.

The 30 and 100 mg/kg/day doses did not affect the selected study parameters. In contrast, the 300 mg/kg dose of MA60 caused a body weight loss of more than 10%. This is in agreement with a study performed with an ethanolic root bark extract of *M. alba* (Yimam et al., 2019). The authors describe an appetite suppression and body weight loss in mice treated orally with daily dosages of 250 and 500 mg/kg. Thus, the 100 mg/kg dose of MA60 fulfills the MTD definition as “The highest dose of a drug or treatment that does not cause unacceptable side effects.”^6^ The MTD for MA60 determined in this study is also in line with previous literature (Dorato and Buckley, 2006) stating that experimentally, the MTD should cause no more than a 10% depression in body weight gain.

Accordingly, 100 mg/kg was applied to get first insights into the potential efficacy of MA60 against influenza virus infection *in vivo*. The body weight loss caused by influenza virus infection, virus replication (virus titer and viral RNA load in lung tissue), and cytokine mRNA induction (IFN-ß, IL-6, IP10, CXCL9 in lung tissue) were selected as readout parameters.

In agreement with the acute toxicity study, the 100 mg/kg dose of MA60 in the efficacy study did not affect body weight confirming the MTD selection. A delayed loss of body weight was observed in MA60-treated, influenza virus-infected mice pointing towards a moderate protective effect. In the presented *in vivo* setting, however, no significant efficacy was obtained. One reason might be an inefficient inhibition of viral replication. Indeed, there was no effect on virus replication (influenza virus titers and number of viral nucleoprotein genome copies) in the lung. This might be based on the antiviral mechanism of action of sanggenons which target the neuraminidase (Grienke et al., 2016; Langeder et al., 2023). The activity of neuraminidase inhibitors in cell-based assays is dependent on a balanced function of the viral hemagglutinin and neuraminidase (Grienke et al., 2012). It could be demonstrated previously that sanggenons dose-dependently inhibited the influenza virus replication in MDCK cells but not in lung bronchial cells (Langeder et al., 2023). A further explanation for the lack of inhibition of influenza virus replication might be an ineffective concentration of bioactive sanggenons in lung tissue.

The analysis of cytokine expression revealed higher amounts of the antiviral IFN-β in MA60-treated, influenza virus-infected mice, compared to placebo-treated, influenza virus-infected mice (*p* = 0.055) at day one after infection. In addition, a highly significant difference (*p* = 0.006) was identified between MA60-treated, influenza virus-infected mice and the placebo-treated, mock-infected group. The more pronounced induction of IFN-β in the presence of MA60 might have contributed to a delayed induction of influenza symptoms as previously reported (Chen et al., 2018). This hypothesis is supported by the delayed body weight loss observed in our efficacy study. Intriguingly, protective effects were recently observed for a white mulberry fruit extract in a rat model, showing protective effects of the applied extract on the fertility of rats treated with the alkylating agent carmustine (Inanc et al., 2022; Ipek et al., 2022). Whether higher amounts of the bioactive sanggenons in lung tissue would reveal a stronger IFN-ß response and whether this is associated with stronger protection against influenza remains to be studied.

In the present study, concentration of bioactive sanggenons C and D was determined in serum and tissue samples and correlated with the administered the doses of MA60. UHPLC-ESI-MS analyses of the *in vitro* bioactive sanggenons C and D revealed serum concentration of less than 1% after one (efficacy study) and three (acute toxicity study) hours of the last oral MA60 administration. Already during dose finding and acute toxicity studies, a high variation in serum concentrations of the selected marker compounds was detected within all three dose groups (30, 100, and 300 mg/kg). No dose dependency could be observed regarding the serum concentrations. This might be due to i) inhomogeneities in the CMC suspension of the MA60 extract, ii) the inter-individual differences in the resorption of sanggenons, or iii) the formation of sanggenon metabolites:

(i) For oral gavage administration, MA60 was suspended in 0.3% carboxymethyl cellulose (CMC) as vehicle. This is based on literature data, where even a higher dose of up to 400 mg/kg of a 70% EtOH extract of *M. alba* root bark (Lim et al., 2013) was previously administered to mice per gavage feeding. In the present study, poor solubility of MA60 prevented the use of higher doses. Thus, the applicability of 0.3% CMC as vehicle could only partly be confirmed for MA60, which could have impacted the homogenous distribution of sanggenons in the oral gavage.
(ii) Inter-individual differences in absorption, distribution, metabolism, and excretions might further affect drug metabolism and contribute to the high variation in serum concentrations of the selected marker compounds.
(ii) Regarding the potential formation of MDAA metabolites, Liu and coworkers found mono- and di-glucuronides as well as hydroxylated derivatives of the *M. alba* constituent kuraridin (prenylated flavonoid) in rat serum (Liu et al., 2018). In the present study, an MS/MS analysis was conducted to also search for metabolites of the selected marker compounds sanggenon C and sanggenon D. The search included (di)glucuronides and hydroxylated metabolites as well as further conjugates such as sulfated metabolites. Interestingly, neither the corresponding masses nor the phase-II-conjugates could be detected in the different serum samples.

A rapid metabolism or excretion of sanggenon C is highly plausible when comparing the quantities determined in mice that were sacrificed 1 hour versus 3 hours after the last application. Here, the average serum concentration decreased from 15 ng/mL to below 2 ng/mL. Hence, no accumulation of MDAAs was observed after oral application.

Intriguingly, in this study, a pronounced difference was observed for extraction recoveries of the isomers sanggenons C and D from the investigated samples: Recovery rates were superior for sanggenon C in serum and in liver samples for sanggenon D. Their difference in stereochemical configuration obviously results in a deviating behavior in the chiral environment of biofluids or tissue samples.

Although for the present study female mice were selected because of their higher susceptibility to influenza virus infection (Robinson et al., 2011), it would be highly worth to analyze the acute toxicity of MA60 also in male mice. Moreover, safety and efficacy are important preclinical parameters (Buckley and Dorato, 2009) which could be more focused on, e.g., by determining the maximum feasible dose (MFD) of MA60.

Overall, the non-significant treatment effect and the determined low serum concentrations of sanggenon C and sanggenon D raise the question about the bioavailability of MA60 after oral application. In future studies, oral administration of MA60 twice a day (BID) or three times a day (TID), intravenous administration, or inhalation administration should be conducted to fully assess the *in vivo* potential of the bioactive sanggenons and to further evaluate their pharmacokinetic parameters.

## Data Availability

The original contributions presented in the study are included in the article/Supplementary Material, further inquiries can be directed to the corresponding authors.

## Footnotes

1
https://www.who.int/news-room/fact-sheets/detail/influenza-seasonal

2
https://www.ema.europa.eu/en/documents/scientific-guideline/ich-guideline-m3r2-non-clinical-safety-studies-conduct-human-clinical-trials-marketing-authorisation_en.pdf

3
https://www.ema.europa.eu/en/documents/scientific-guideline/ich-q-2-r1-validation-analytical-procedures-text-methodology-step-5_en.pdf

4
https://www.ema.europa.eu/en/documents/scientific-guideline/ich-s-7-b-nonclinical-evaluation-potential-delayed-ventricular-repolarization-qt-interval_en.pdf

5
https://www.ema.europa.eu/en/ich-m3-r2-non-clinical-safety-studies-conduct-human-clinical-trials-pharmaceuticals-scientific

6
https://www.cancer.gov/publications/dictionaries/cancer-terms/def/maximum-tolerated-dose
