# Supplementary material for: Correlation of bioactive marker compounds of an orally applied Morus alba root bark extract with toxicity and efficacy in BALB/c mice
Source: Front Pharmacol. 2023 Dec 8;14:1193118. doi: 10.3389/fphar.2023.1193118 (PMC10739329; doi:10.3389/fphar.2023.1193118)
Supplement: Supplementary file 1 [file DataSheet1.PDF]

Supplementary Material

Correlation of bioactive marker compounds of an orally applied *Morus alba* root bark extract with toxicity and efficacy in BALB/c mice

Julia Langeder[1,2], Mirijam Koch[3], Hannes Schmietendorf[4], Ammar Tahir[2], Ulrike Grienke[2], Judith M. Rollinger[2†]*, Michaela Schmidtke[4†*]

[†]These authors share senior authorship

***Correspondence:**
Michaela Schmidtke
michaela.schmidtke@med.uni-jena.de

Judith M. Rollinger
judith.rollinger@univie.ac.at

1 Supplementary Figures and Tables

1.1 Supplementary Figures

Supplementary Figure 1. (A) Photographs of MA60 (30, 100 or 300 mg/kg) dissolved in aqueous 0.3% carboxymethyl cellulose (CMC) and (B) experimental groups of the dose-finding study.

(A) Photographs of MA60 suspension

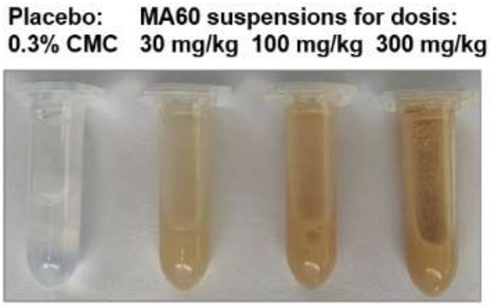

(B) Experimental groups

Treatment	MA60 dosis (mg/kg)	Number of mice
Placebo (0.3% CMC)	0	5
MA60	300	5
	100	5
	30	5
**Overall**		**20**

Supplementary Figure 2. Similarity of MS/MS spectra of (A) sanggenon C in comparison to serum samples #38 and #64 and (B) sanggenon D in comparison to liver samples #64 and #65.

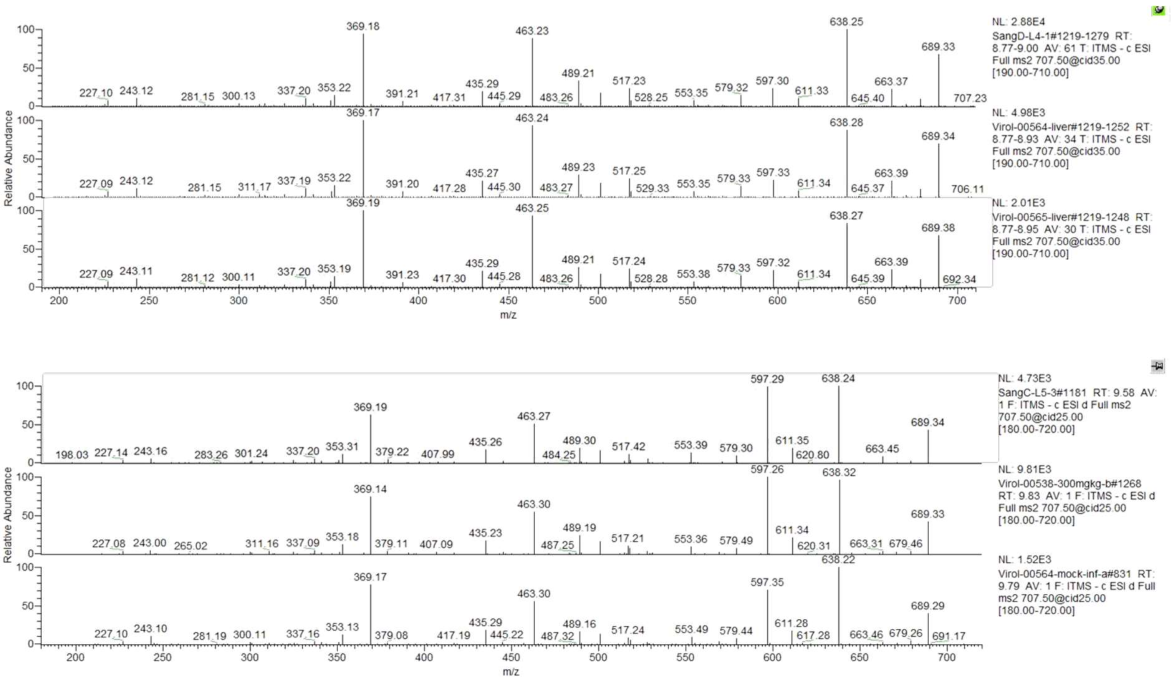

Supplementary Figure 3: Photographs of hematoxylin-eosin-stained lung sections of day 1 p.i. Lung section of a (A) placebo-treated, mock-infected, (B) placebo-treated, influenza virus-infected, (C) MA60-treated, mock-infected, and (D) MA60-treated, influenza virus-infected BALB/c mouse is shown for example.

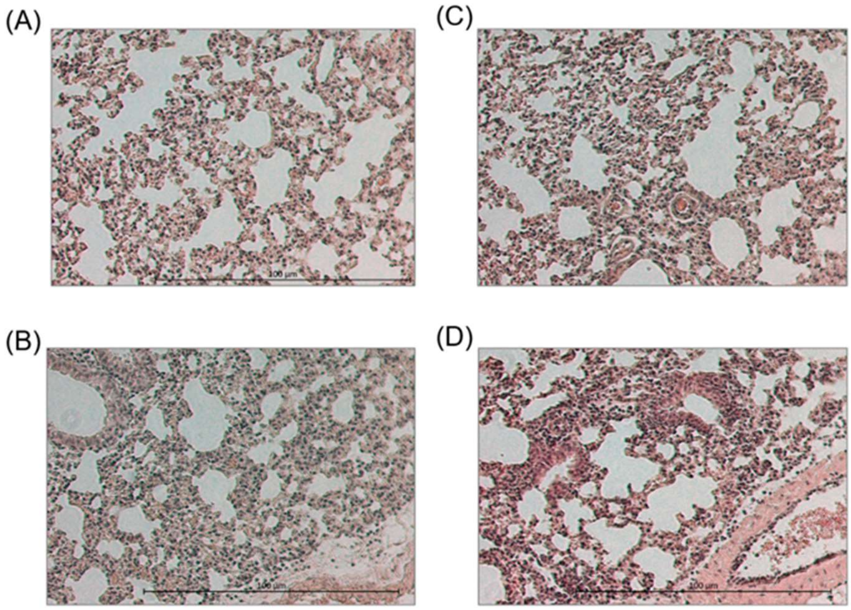

1.2 Supplementary Tables

Supplementary Table 1: Experimental groups of the efficacy study.

Treatment	Infection	Days p.i.	Number of mice
Placebo (0.3% CMC)	No	1	4
		7	4
	Influenza virus	1	5
		7	10
MA60 (100 mg/kg in 0.3% CMC)	No	1	4
		7	4
	Influenza virus	1	5
		7	10
**Overall**			**46**

Supplementary Table 2: Primer pairs

Target gene	Primer sequence
Murine_GAPDH_forward	GCA TCT TCT TGT GCA GTG CC
Murine_GAPDH_reverse	ATG AAG GGG TCG TTG ATG GC
Murine_IP-10_forward	CCA AGT GCT GCC GTC ATT TT
Murine_IP-10_reverse	AGC TTC CCT ATG GCC CTC AT
Murine_IL-6_forward	CCC CAA TTT CCA ATG CTC TCC
Murine_IL-6_reverse	CGC ACT AGG TTT GCC GAG TA
Murine_IFNß_forward	TGC CTT TGC CAT CCA AGA GA
Murine_IFNß_reverse	TGT CTG CTG GTG GAG TCC ATC
Murine_CXCL9_forward	TAG AGC CCC TGC ACA CAT TG
Murine_CXCL9_reverse	CGA AAG CTA CGT GGG AGG TT
Murine_Eif2ak2_forward	AGT TAC CAT AGG GCA AGC CT
Murine_Eif2ak2_reverse	CAA GAA ACG CTG CAG CCA AA

Supplementary Table 3: Temperature profile for NP-gene quantification:

Temperature	Time	Number of cycles
95° C	5 min	
95° C	10 sec	37 cycles
55° C	30 sec	
72° C	120 sec	
60° C – 95° C	Melting curve analysis	
20° C	10 sec	

Supplementary Table 4: Temperature profile for cytokine analysis

Temperature	Time	Number of cycles
95° C	5 min	
95° C	10 sec	35 cycles
59° C	30 sec	
72° C	120 sec	
60° C – 95° C	Melting curve analysis	
20° C	10 sec	

Supplementary Table 5: Statistical analysis of the percentage of daily body weight in the acute toxicity study with MA60. The applied test with p values are summarized.

Test/results of ANOVA		Day of treatment 2	3	4	5	6	7
Normality test (Shapiro-Wilk)		passed (p=0.202)	passed (p=0.081)	passed (p=0.793)	passed (p=0.247)	passed (p=0.704)	passed (p=0.829)
Equal Variance Test (Brown-Forsythe)		passed (p=0.325)	failed (p<0.05)	passed (p=0.062)	passed (p=0.364)	passed (p=0.140)	failed (p<0.05)
Difference in the mean values of treatment groups		**not significant (p=0.106)**	**not significant (p=0.262)**	significant (p=0.029)	**not significant (p=0.076)**	**not significant (p=0.063)**	**not significant (p=0.056)**
**Comparison**			**Kruskall-Wallis ANOVA on Ranks**	**pairwise multiple comparison procedure (Holm-Sidak method)**			**Kruskall-Wallis ANOVA on Ranks**
Placebo	30 mg/kg			0.664			
Placebo	100 mg/kg			0.748			
Placebo	300 mg/kg			0.030			
30 mg/kg	100 mg/kg			0.720			
30 mg/kg	300 mg/kg			0.166			
100 mg/kg	300 mg/kg			0.103			

Supplementary Table 6: Body and organ weights after 7 days of placebo (0.3% CMC) and MA60 treatment (30, 100 or 300 mg/kg, once per day).

Mouse	Treatment	Body weight (g)	Organ weight (mg)			
			Lung	Spleen	Heart	Liver
**1**	Placebo	19.50	169	94	94	941
**2**		19.12	179	89	85	901
**3**		17.84	153	85	83	825
**4**		21.14	150	79	73	927
**5**		18.84	145	87	92	1150
**Mean**		**19.40**	**162.75**	**86.75**	**83.75**	**898.50**
**SD**		**1.20**	**14.25**	**5.50**	**8.32**	**121.07**
**6**	MA60	18.63	140	85	95	894
**7**	(30 mg/kg)	18.22	154	89	79	995
**8**		19.71	166	107	80	1010
**9**		18.38	143	112	72	1065
**10**		18.57	145	95	84	894
**Mean**		**18.74**	**150.75**	**98.25**	**81.50**	**991.00**
**SD**		**0.59**	**10.55**	**11.57**	**8.46**	**75.48**
**11**	MA60	20.71	151	91	86	1131
**12**	(100 mg/kg)	18.52	131	83	76	1045
**13**		19.30	144	80	82	943
**14**		18.72	137	86	77	889
**15**		17.03	105	75	67	939
**Mean**		**18.86**	**133.60**	**83.00**	**77.60**	**989.40**
**SD**		**1.33**	**17.66**	**6.04**	**7.16**	**97.36**
**16**	MA60	17.68	148	90	82	852
**17**	(300 mg/kg)	18.16	143	77	89	812
**18**		16.98	153	87	80	896
**19**		18.83	142	84	83	845
**20**		17.90	150	73	75	926
**Mean**		**17.91**	**146.50**	**84.50**	**83.50**	**851.25**
**SD**		**0.68**	**4.66**	**7.05**	**5.07**	**44.87**

Supplementary Table 7: Statistical analysis of the percentage of daily body weight in the efficacy study with MA60. The applied tests and calculated p values are summarized.

Test/results of ANOVA		Days p.i.(of treatment) 1(2)	2(3)	3(4)	4(5)	5(6)	6(7)	7
Normality test (Shapiro-Wilk)		failed (p<0,050)	failed (p<0,050)	failed (p<0,050)	passed (p=0,188)	failed (p<0,050)	failed (p<0,050)	failed (p<0,050)
Equal Variance Test (Brown-Forsythe)					passed (p=0,110)			
Difference in the mean values of treatment groups		not significant (p=0,233)	significant (p=0,009)	significant (p<0,001)	significant (p<0,001)	significant (p<0,001)	significant (p<0,001)	significant (p<0,001)
Comparison		Kruskall-Wallis ANOVA on Ranks	all pairwise multiple comparison procedure (Dunn´s method)	all pairwise multiple comparison procedure (Dunn´s method)	pairwise multiple comparison procedure (Holm-Sidak method)	all pairwise multiple comparison procedure (Dunn´s method)	all pairwise multiple comparison procedure (Dunn´s method)	all pairwise multiple comparison
Placebo-treated+mock-infected	MA60-treated+mock-infected		1.000	1.000	0.973	1.000	1.000	1.000
Placebo-treated+mock-infected	Placebo-treated+IAV-infected		0.020	0.002	<0,001	0.004	0.003	0.002
Placebo-treated+mock-infected	MA60-treated+IAV-infected		1.000	0.022	0.004	0.038	0.105	0.087
MA60-treated+mock-infected	Placebo-treated+IAV-infected		0.095	0.027	<0,001	0.015	0.004	0.008
MA60-treated+mock-infected	MA60-treated+IAV-infected		1.000	0.172	0.004	0.121	0.140	0.212
Placebo-treated+IAV-infected	MA60-treated+IAV-infected		0.204	1.000	0.267	1.000	0.794	0.878

Supplementary Table 8: Statistical analysis of the cytokine mRNA levels in the efficacy study with MA60. The applied test and calculated p values are summarized.

IL-6

		Days p.i.	
**Test/results of ANOVA**		**1**	**7**
**Normality test (Shapiro-Wilk)**		passed (p=0.309)	passed (p=0.550)
**Equal Variance Test (Brown-Forsythe)**		passed (p=0.705)	passed (p=0.679)
**Difference in the mean values of treatment groups**		**significant (p=0.021)**	**significant (p<0.001)**
**Comparison**		**all pairwise multiple comparison procedure (Bonferroni t-test))**	**all pairwise multiple comparison procedure (Bonferroni t-test))**
**Placebo-treated+mock-infected**	**MA60-treated+mock-infected**	1.000	0.211
**Placebo-treated+mock-infected**	**Placebo-treated+IAV-infected**	1.000	**<0.001**
**Placebo-treated+mock-infected**	**MA60-treated+IAV-infected**	**0.030**	**<0.001**
**MA60-treated+mock-infected**	**Placebo-treated+IAV-infected**	1.000	**0.010**
**MA60-treated+mock-infected**	**MA60-treated+IAV-infected**	0.062	**0.008**
**Placebo-treated+IAV-infected**	**MA60-treated+IAV-infected**	0.314	1.000

IP10

		Days p.i.	
**Test/results of ANOVA**		**1**	**7**
**Normality test (Shapiro-Wilk)**		passed (p=0.927)	passed (p=0.622)
**Equal Variance Test (Brown-Forsythe)**		passed (p=0.155)	passed (p=0.829)
**Difference in the mean values of treatment groups**		**significant (p=0.011)**	**significant (p<0.001)**
**Comparison**		**all pairwise multiple comparison procedure (Bonferroni t-test))**	**all pairwise multiple comparison procedure (Bonferroni t-test))**
**Placebo-treated+mock-infected**	**MA60-treated+mock-infected**	1.000	1.000
**Placebo-treated+mock-infected**	**Placebo-treated+IAV-infected**	0.509	**<0.001**
**Placebo-treated+mock-infected**	**MA60-treated+IAV-infected**	**0.015**	**<0.001**
**MA60-treated+mock-infected**	**Placebo-treated+IAV-infected**	1.000	**<0.001**
**MA60-treated+mock-infected**	**MA60-treated+IAV-infected**	**0.039**	**<0.001**
**Placebo-treated+IAV-infected**	**MA60-treated+IAV-infected**	0.433	1.000

CXCL9

		Days p.i.	
**Test/results of ANOVA**		**1**	**7**
**Normality test (Shapiro-Wilk)**		passed (p=0.927)	passed (p=0.622)
**Equal Variance Test (Brown-Forsythe)**		passed (p=0.155)	passed (p=0.829)
**Difference in the mean values of treatment groups**		**significant (p=0.011)**	**significant (p<0.001)**
**Comparison**		**all pairwise multiple comparison procedure (Bonferroni t-test))**	**all pairwise multiple comparison procedure (Bonferroni t-test))**
**Placebo-treated+mock-infected**	**MA60-treated+mock-infected**	1.000	1.000
**Placebo-treated+mock-infected**	**Placebo-treated+IAV-infected**	0.509	**<0.001**
**Placebo-treated+mock-infected**	**MA60-treated+IAV-infected**	**0.006**	**<0.001**
**MA60-treated+mock-infected**	**Placebo-treated+IAV-infected**	1.000	**<0.001**
**MA60-treated+mock-infected**	**MA60-treated+IAV-infected**	**0.041**	**<0.001**
**Placebo-treated+IAV-infected**	**MA60-treated+IAV-infected**	0.465	1.000

IFN beta

		Days p.i.	
**Test/results of ANOVA**		**1**	**7**
**Normality test (Shapiro-Wilk)**		passed (p=0.545)	passed (p=0.524)
**Equal Variance Test (Brown-Forsythe)**		passed (p=0.392)	passed (p=0.645)
**Difference in the mean values of treatment groups**		**significant (p=0.007)**	**not significant (p=0.168)**
**Comparison**		**all pairwise multiple comparison procedure (Bonferroni t-test))**	
**Placebo-treated+mock-infected**	**MA60-treated+mock-infected**	0.517	
**Placebo-treated+mock-infected**	**Placebo-treated+IAV-infected**	1.000	
**Placebo-treated+mock-infected**	**MA60-treated+IAV-infected**	**0.006**	
**MA60-treated+mock-infected**	**Placebo-treated+IAV-infected**	1.000	
**MA60-treated+mock-infected**	**MA60-treated+IAV-infected**	0.168	
**Placebo-treated+IAV-infected**	**MA60-treated+IAV-infected**	0.055	

Supplementary Table 9. Quantitative results of sanggenon C in serum samples and sanggenon D in liver and lung samples for the acute toxicity study in which 30 mg/kg, 100 mg/kg, and 300 mg/kg extract (MA60) were applied orally and the efficacy study with 100 mg/kg body weight, respectively.

Group	Sample	quantified amount of sanggenon C serum [ng/mL]	± SD	quantified amount of sanggenon D liver [ng/mL]	± SD	quantified amount of sanggenon D lung [ng/mL]	± SD
Mock	MTBE blank	n.f.	-	n.d.	-	n.f.	-
	MeOH blank	n.f.	-	n.f.	-	n.f.	-
	23	n.f.	-	n.d.	-	n.f.	-
	24	n.d.	-	n.f.	-	n.f.	-
	25	n.d.	-	n.f.		n.f.	-
30 mg/kg	26	n.f.	-	n.f.	-	n.f.	-
	27	4.97	0.73	n.f.	-	n.f.	-
	28	9.10	0.04	n.f.	-	n.f.	-
	29	n.f.	-	n.f.	-	1,005.12	363.45
	30	n.f.	-	n.f.	-	n.f.	-
100 mg/kg	31	9.28	0.22	n.f.	-	n.f.	-
	32	31.53	0.03	34.18	3.01	2,980.27	679.59
	33	11.17	1.02	n.f.	-	343.27	79.39
	34	13.02	0.54	n.f.	-	n.f.	-
	35	7.94	0.18	26.97	0.44	10,306.71	411.57
300 mg/kg	36	25.74	0.17	70.13	19.14	n.f.	-
	37	21.21	0.25	89.18	9.23	n.f.	-
	38	227.26	0.14	979.27	9.95	n.f.	-
	39	24.13	3.14	996.87	54.59	n.f.	-
	40	103.00	7.16	447.53	18.69	n.f.	-
Mock treated	41	n.f.	-	n.f.	-	n.f.	-
	42	n.f.	-	n.f.	-	n.f.	-
	43	n.f.	-	n.f.	-	n.f.	-
	44	n.f.	-	n.f.	-	n.f.	-
	45*	n.f.	-	n.f.	-	n.f.	-
	46*	n.f.	-	n.f.	-	n.f.	-
	47*	n.f.	-	n.f.	-	n.f.	-
	48*	n.f.	-	n.f.	-	n.f.	-
100 mg/kg	64	124.22	0.08	2,344.66	78.13	n.f.	-
	65	34.56	0.72	1,278.95	60.76	n.f.	-
	66	46.70	0.96	885.31	24.79	n.f.	-
	67	8.00	0.23	330.30	6.01	n.f.	-
	68*	2.77	0.02	n.f.	-	n.f.	-
	69*	2.62	0.09	n.f.	-	n.f.	-
	70*	n.f.	-	n.f.	-	n.f.	-
	71*	n.f.	-	n.f.	-	n.f.	-

* Sacrificed 24 h after last application (once daily for seven days)
n.d. … not determined; n.f. … not found
